# The effect of balanced versus non-balanced fluid replacement on urinary biomarkers in patients undergoing colectomy: a prospective observational pilot study

**DOI:** 10.1186/2197-425X-3-S1-A237

**Published:** 2015-10-01

**Authors:** G Rooney, B Kelly, T Konrad, M Scully, J Bates

**Affiliations:** Department of Anaesthesia and Intensive Care Medicine, University Hospital Galway, Galway, Ireland

## Intr

Several studies have suggested an association between chloride rich fluids and acute kidney injury (AKI) in perioperative and critically ill patients [1]. The mechanism of injury has not been fully described but renal haemodynamic effects and a change in oxidative stress have been hypothesised. Urinary Cystatin C (uCysC) is a sensitive and early marker of renal tubular injury [2]. Levels of uCysC correlate well with adverse clinical outcomes such as AKI and mortality. Urinary neutrophil gelatinase-associated lipocalin (uNGAL) has been shown to be an excellent predictor of acute renal injury in paediatric and adult cardiac surgery [3] but has not previously been studied in non-cardiac surgery.

## Objectives

To assess the effect of balanced versus non-balanced fluid replacement in peri-operative patients using uCysC and uNGAL.

## Methods

We performed a prospective observational pilot study on patients with normal baseline renal function undergoing elective colectomy. Patients received either Hartmann´s Solution or 0.9% Saline intra-operatively according to a stroke volume variation based protocol. Urine samples were taken at baseline, on completion of surgery, 4 hours and 12 hours post completion. Baseline and post-operative day 1 creatinine levels were measured.

## Results

Twenty patients (n = 20) were recruited for this pilot study,10 in each group. There was no significant difference in gender distribution, mean age, mean pre-op pH, mean baseline NGAL and Cystatin between the groups. Patients receiving 0.9% Saline had a higher post-op chloride with a mean difference of 4.4 mmol (95% 0.3-7.98, p = 0.019). pH was lower in the saline group (7.33 vs 7.37) but this was not statistically significant (p = 0.10). No significant difference was seen in intra-operative fluid volume and urine output at 4 hours. Patients receiving 0.9% Saline had a higher urine output at 12 hours with mean difference of 239 ml (95%CI 61.04-416.97, p = 0.011). There was no significant difference between pre and post op uNGAL or uCysC at 4 and 12 hours in both groups. There was no significant difference in level of uNGAL or uCysC between the two groups at all time-points.

## Conclusions

Patients treated with 0.9% Saline but not Hartmann's Solution developed hyperchloremia but this did not result in a rise in uNGAL or uCysC. Patients undergoing elective colectomy do not develop a rise in uNGAL or uCysC as a result of the operative process, unlike those undergoing cardiopulmonary bypass.

## Grant Acknowledgment

Research was facilitated by a grant from the NUI Galway Millennium Research Fund.

## References

[1] Burdett (2012). Perioperative buffered versus non-buffered fluid administration for surgery in adults. Cochrane Database Syst Rev.

[2] Nejat (2010). Rapid detection of acute kidney injury by plasma cystatin C in the intensive care unit. Nephrol Dial Transplant.

[3] Koyner (2010). Urinary biomarkers in the clinical prognosis and early detection of acute kidney injury. Clin J Am Soc Nephrol.

